# Association study of six candidate genes with major depressive disorder in the North-Western population of Pakistan

**DOI:** 10.1371/journal.pone.0248454

**Published:** 2021-08-19

**Authors:** Naqash Alam, Sadiq Ali, Nazia Akbar, Muhammad Ilyas, Habib Ahmed, Arooj Mustafa, Shehzada Khurram, Zeeshan Sajid, Najeeb Ullah, Shumaila Qayyum, Tariq Rahim, Mian Syed Usman, Nawad Ali, Imad Khan, Khola Pervez, BiBi Sumaira, Nasir Ali, Nighat Sultana, Adeel Yunus Tanoli, Madiha Islam

**Affiliations:** 1 School of Basic Medical Sciences, Health Science Center, Xi’an Jiaotong University, Xi’an, Shaanxi, China; 2 Department of Biotechnology and Genetic Engineering, Hazara University, Mansehra, Pakistan; 3 Centre for Omic Sciences, Islamia College University, Peshawar, Pakistan; 4 Department of Biochemistry, Hazara University, Mansehra, Pakistan; National Institutes of Health, UNITED STATES

## Abstract

People around the world are currently affected by Major Depressive Disorder (MDD). Despite its many aspects, symptoms, manifestations and impacts, efforts have been made to identify the root causes of the disorder. In particular, genetic studies have concentrated on identifying candidate genes for MDD and exploring associations between these genes and some specific group of individuals. The aim of this research was to find out the association between single nucleotide polymorphisms in 6 candidate genes linked to the neurobiology of major depressive disorder in the North-Western population of Pakistan. We performed a case-control analysis, with 400 MDD and 232 controls. A trained psychiatrist or clinical psychologists evaluated the patients. Six polymorphisms were genotyped and tested for allele and genotype association with MDD. There were no statistical variations between MDD patients and healthy controls for genotypic and allelic distribution of all the polymorphisms observed. Thus, our analysis does not support the major role of these polymorphisms in contributing to MDD susceptibility, although it does not preclude minor impact. The statistically significant correlation between six polymorphisms and major depressive disorder in the studied population was not observed. There are inconsistencies in investigations around the world. Future research, including GWAS and association analysis on larger scale should be addressed for further validation and replication of the present findings.

## Introduction

Major depression is the most prevalent psychiatric disorder; about 450 million people are affected and the predominant cause of the morbidity and disability worldwide [1]. Every year, 6–7% of the population and 16% of people are affected by depression during their lives [2,3]. In a given year, according to the World Health Organization, 9% of women and 5% of men have developed depressive disorder [4]. It is accompanied by the following symptom; sadness, lack of energy and interest, mood and behavior changes and abnormal circadian rhythms. Major depressive disorder is very sever and heterogeneous and can lead to suicide when it not treated [4,5]. In the etiology of major depression, genetic factors clearly play a significant role, as shown by family and twined studies that indicate heritability estimates of 17% to 75% on average at 37% [6–8].

In the development of psychiatric disorders, both genetic disposition and environmental factors are significant, especially severe psychiatric disorders such as major depression [9]. It is because the cumulative contribution of genetic influence might be stronger for those severe mental disorders [10], and a range of environmental factors seem to result in an increased proportion of cases of serious mental illness [9,11].

Until recently, the major depression genetics research was primarily focused on candidate genes, or genes that were hypothesized to be involved in the neurobiology of major depression. The most commonly studied candidate genes were those regulating the neurotransmission of serotonin (5-HT) and dopamine (DA), considering the potential role of these neurotransmitters in the pathophysiology of depression and the fact that they are targets of antidepressants drugs [12–14].

Genetic variations are presumed to have only minor effects on the overall risk of disease and multiple genetic factors combined with the environmental factors [15,16]. The basic concept of genetic association studies is that a genetic variant is investigated in both cases and control [16]. Jia et al, 2011 reported 151 candidate genes whose variations were related to the development of MDD [17]. Howard *et al*, 2019 identified 102 independent variations, 269 genes, and 15 gene-sets linked with major depression, including both gene(s) and gene pathways that are involved with synaptic structures and neurotransmission [18]. Unfortunately, the majority of studies on candidate genes were underpowered and the results were rarely duplicated. The availability of DNA microarrays has recently allowed for genome-wide association studies (GWAS) which are not based on previous hypotheses. A million or more variants can be analyzed using the GWAS approach around the whole genome. The major objective of these genetic association studies is to improve diagnosis, prevention, and treatment by a nuanced understanding of the genetic basis of the disease [19]. In this study, we carried out gene-based as well as single marker analyses in six genes to identify genetic variations that could affect the risk of MDD in Pakistani population.

## Materials and methods

### Subjects

A case-control study was conducted to investigate the association of gene polymorphisms with 400 patients and 232 controls with major depressive disorder; the diagnosis of MDD was made based on the Diagnostic and Statistical Manual of Mental Disorders—Fourth Edition (DSM-IV). This study included patients who had been diagnosed with MDD by at least two qualified psychiatrists. The first psychiatric specialist interviewed the patients. When the patient was diagnosed with MDD, according to DSM-IV guideline, another psychiatric specialist was employed to confirm the diagnosis. The 17-item Hamilton Rating Scale for Depression (HAMD-17) was used to assessed the severity of depression. The research included only subjects with a minimum score of 18 on HAMD-17. The study excluded patients with a history of neurological illness or other psychiatric disorders. Patients with serious organic conditions were excluded to prevent cases with secondary depression [S1 and S2 Tables]. The designed study was approved by the Ethical Committee of the Hazara University Mansehra, KP Province. The written informed consent was signed from all the subjects and/or their parents. Individual information on occupation, ethnicity, co-morbidities, and level of education was recorded.

### Selection of SNPs

The candidate genetic SNPs met at least two from the following criteria: (1) The SNPs must have been linked to MDD or a depressive episode in the previous; (2) The SNPs are located within the genes in the intronic or exonic region; (3) A minor allele frequency of *>*5% in the north-western population. The polymorphisms were selected from the public domain of the National Center for Biotechnology Information’s (NCBI dbSNP) database of SNPs, which can be accessed at http://www.ncbi.nlm.nih.gov/snp (Bethesda, Montgomery County, MD, USA).

### DNA extraction and nucleotide sequences

Each participant was deliberated to obtain 5 ml of whole blood and placed in the anticoagulant EDTA tubes. Before processing, samples were saved at −20°C. The standard phenol chloroform method was then used to extract genome DNA. The polymerase chain reaction (PCR) method was used for DNA amplification. The primers were designed by using Primer 5.0 software for PCR amplification, and their specificity was validated using NCBI BLASTN (http://www.ncbi.nlm.nih.gov/BLAST/) Table 1. PCR amplification was performed with a reaction volume of 25 μl containing 2.5 μl buffer 109 (Tiangen, Beijing, China), 200 μM each dNTP, 0.4 μM primers each, and 1.0 unit of Taq DNA polymerase (Tiangen, Beijing, China) and 60 ng of genomic DNA. Conditions used for PCR amplification were denaturation at 95°C for 10 min, followed by 35 cycles of 95°C for 30 s, 54–56°C for 30 s, and 72°C for 30 s, followed by a final extension at 72°C for 12 min. Table 3 contains primer sequences used for amplification of DNA fragments containing polymorphisms. Sanger sequencing analysis was performed by Macrogen Korea from amplified PCR products, for comparative basis, where the patient’s electropherogram was compared against an electropherogram from healthy/control samples. The observed differences between the two groups was recorded and analyzed. DNA sequence was analyzed using ClustalW multiple sequence alignment (Bioedit, v7.0.9; available at http://www.mbio.ncsu.edu/BioEdit/page2.html).

**Table 1:** Primers for PCR amplification.

Gene	SNP ID (GRCh38)	Primer Sequence	Annealing temperature	Fragment Size
*GSK3B*	rs334535	F 5’-ACAGCCAGCAAGATGCATAG-3’	54°C	418bp
		R 5’-AACCACACCACAGCACTTCA-3’		
*TPH1*	rs1800532	F 5’- GTTTTTCCATCCGTCCTGTG-3’	54°C	442bp
		R 5’- CTGTTTCCCCCACTGGAATA-3’		
*TPH2*	rs7305115	F 5’-CCTGGAAACCGTCATTTGAG-3’	54°C	436bp
		R 5’-TGGAGGTAAAACAGGGCCTA-3’		
*DAT1*	rs40184	F 5’-AAGTCGCTGGGGTACAATCT-3’	54°C	440bp
		R 5’-TTCGTGTCTCTCCCATTGCA-3’		
*SLC6A15*	rs1545843	F 5’-CTGCAGGTCTCAGCATTTCA-3’	54°C	439bp
		R 5’-CAAACCAGGATCCAGTCAAG-3’		
*BDNF*	rs6265	F 5’-TTCTCCCTACAGTTCCACCA-3’	56°C	545bp
		R 5’-GGGACCTTTTCAAGGACTGT-3’		

### Statistical analysis

The Hardy Weinberg equilibrium was assayed using the Chi square test from the Hardy–Weinberg equilibrium for association in the entire sample, and both in depressed individuals and controls. The chi-squared test was used to compare genotypes and allele frequencies between groups. The significance level for all statistical tests was 0.05.

## Results

The genotype frequency was found to be in Hardy–Weinberg equilibrium [S3 and S4 Tables] in the entire study samples in both cases and controls (*P*>0.05 in all cases). Tables 2–4 shows the studied genes, data for genotype and allele frequencies. As shown in Tables 3 and 4, there were no significant differences between the case groups and the control group in the allele or genotype frequencies of each polymorphism. To study the association between MDD and polymorphisms of the GSK3B gene, we found genotype (χ2 = 1.16; P = 0.560) and allele (χ2 = 0.78; P = 0.376) frequencies of SNP rs334535 showed no significant difference between patients and control groups. Based on the calculated Chi square and P value for the genotype frequencies of rs1800532 of TPH1 gene, showed no significant differences amongst the MDD patients and controls group, genotype (χ2 = 10.6; *P =* 0.005) allele (χ2 = 6.40; *P =* 0.011). The genotype distributions of rs7305115 of TPH2 gene, (χ2 = 16.6; *P* = 0.000) and allele frequencies (χ2 = 8.46; *P* = 0.004) were not significantly different between MDD and normal controls. Furthermore, there were no significant difference in the genotype distribution between MDD and normal controls (χ2 = 13.6; *P* = 0.001) or allele frequency (χ2 = 7.05; *P* = 0.008) of rs40184 of DAT1 gene. There was no difference between the MDD and without depression in genotype distribution (χ2 = 13.5; *P* = 0.001) or allele frequency (χ2 = 7.23; *P* = 0.007) of the rs1545843 polymorphism of SLC6A15 gene. There was also no significant difference in the genotype distribution (χ2 = 19.3; *P* = 6.554) or allele frequency (χ2 = 40.13; *P* = 2.377) of the rs6265 of BDNF gene between MDD and controls. The frequency of the A allele of all SNPs was higher in patients than in controls. Patient’s history, family history and demographic data of patients and controls are given in Table 5.

**Table 2:** Characteristics of the studied polymorphisms.

Gene	SNP ID	Position	Polymorphism	Localization
*GSK3B*	rs334535	Chr:3:120073457	A/G	Intron
*TPH1*	rs1800532	Chr:11:18026269	A/C	Intron
*TPH2*	rs7305115	Chr:12:7197908	A/G	Exon
*DAT1*	rs40184	Chr:5:1394962	A/G	Intron
*SLC6A15*	rs1545843	Chr:12:84170289	A/G	Intron
*BDNF*	rs6265	Chr:11:27658369	G/A	Exon

**Table 3:** Genotypic frequency of the polymorphisms in patients of MDD and control groups.

Genotype Frequencies	SNP,s	Cases		Controls		*χ2*	*Df*	*P*
		*N*	%	*N*	%			
*GSK3B*	rs334535							
	AA	21	24.7	17	42.5	1.16	2	0.560
	AG	46	54.1	10	25.0			
	GG	18	21.2	13	32.5			
*TPH1*	rs1800532							
	AA	25	50.0	11	36.6	10.6	2	0.005
	AC	15	30.0	9	30.0			
	CC	10	20.0	10	33.3			
*TPH2*	rs7305115							
	AA	32	42.6	23	46.0	16.6	2	0.000
	AG	26	34.6	12	24.0			
	GG	17	22.6	15	30.0			
*DAT1*	rs40184							
	AA	25	50.0	13	40.6	13.6	2	0.001
	AG	12	24.0	11	34.3			
	GG	13	26.0	8	25.0			
*SLC6A15*	rs1545843							
	AA	22	44.0	15	50.0	13.5	2	0.001
	AG	15	30.0	8	26.6			
	GG	13	26.0	7	23.3			
*BDNF*	rs6265							
	GG	53	58.8	25	50.0	19.3	2	6.554
	GA	22	24.4	15	30.0			
	AA	15	16.6	10	20.0			

**Table 4:** Allelic frequency of the polymorphisms in patients of MDD and control groups.

Allelic frequencies	SNP,s	Cases		Controls		*χ2*	*Df*	*P*
		*N*	%	*N*	%			
*GSK3B*	rs334535							
	A	88	51.7	44	55.0	0.78	1	0.376
	G	82	48.2	36	45.0			
*TPH1*	rs1800532							
	A	65	65.0	31	51.6	6.40	1	0.011
	C	35	35.0	29	48.3			
*TPH2*	rs7305115							
	A	90	60.0	58	58.0	8.46	1	0.004
	G	60	40.0	42	42.0			
*DAT1*	rs40184							
	A	62	62.0	37	57.8	7.05	1	0.008
	G	38	38.0	27	42.1			
*SLC6A15*	rs1545843							
	A	59	59.0	38	63.3	7.23	1	0.007
	G	41	41.0	22	36.6			
*BDNF*	rs6265							
	G	128	71.1	65	65.0	40.13	1	2.377
	A	52	28.8	35	35.0			

**Table 5:** Demographic and clinical characteristics of patients with MDD and healthy controls.

Variables	Cases		Controls		Totals	
	n = 400	%	n = 232	%	n = 632	%
**Gender**						
Male	197	49.3	121	52.2	318	50.3
Female	203	50.8	111	47.8	314	49.7
**Age**						
15–30	175	43.8	101	43.5	276	43.7
31–60	189	47.3	110	47.4	299	47.3
61 and above	36	9.0	21	9.1	57	9.0
**Weight**						
30–50	38	9.5	24	10.3	62	9.8
51–80	330	82.5	193	83.2	523	82.8
81 and above	32	8.0	15	6.5	47	7.4
**Marital Status**						
Unmarried	102	25.5	56	24.1	158	25.0
Married	280	70.0	167	72.0	447	70.7
Divorced	16	4.0	7	3.0	23	3.6
Widow	2	0.5	2	0.9	4	0.6
**Education**						
Illiterate	176	44.0	101	43.5	277	43.8
Primary	66	16.5	40	17.2	106	16.8
Grade 10	65	16.3	36	15.5	101	16.0
Grade 12	51	12.8	35	15.1	86	13.6
Undergraduate	17	4.3	7	3.0	24	3.8
Post graduate	25	6.3	13	5.6	38	6.0
**Occupation**						
Employed	50	12.5	32	13.8	82	13.0
Unemployed	302	75.5	174	75.0	476	75.3
Students	48	12.0	26	11.2	74	11.7

## Discussion

The advancement of genetic studies and knowledge of the specific genes, physiological interference responsible in the onset of MDD very limited and is still unclear. Most of the candidate studies only focusing on gene involvement in neurotransmitters circuits or stress reactions which only provided suggestive results although not definitive results. One of the explanations may be phylogenetic nature of the disorder and the limited impact of hypothetically high number of involved loci. In addition, MDD tends to be less homogeneous across population then other psychiatric disorder as bipolar disorder and schizophrenia [20]. To robust the association of such association’s reports independent studies on samples is necessary. In current study, samples of general population aimed to replicate as previously reported association between candidate gene and MDD of North-Western population (Khyber Pakhtunkhwa of Pakistan). Best to our knowledge it is first ever study examining the association of single SNPs in six genes candidate (*GSK-3Β*, *TPH1*, *TPH2*, *DAT1*, *SLC6A15*, *and BDNF*) in North-Western population. 400 MDD patients samples were compared with 232 control samples; all SNP’s were calculated through HWE. The GSK-3β gene maps to chromosome 13q13.319 and is located in the same region as the dopamine receptor D3 gene, D3 receptors were localized to limbic areas and may related to cognitive, emotional and endocrine functions [21]. Several lines of evidence show GSK-3Β is a good candidate gene for MDD susceptibility. As to date not all studies show that GSK-3Β itself is responsible for MDD but with unique clinical characteristics [22]. In current study we investigated differences in GSK-3Β gene polymorphisms among patients with MDD, according to individual genetic differences. No difference in the rs334535 polymorphism was found between MDD and normal controls. Our current findings are similar to that of a previous report which was conducted to know genetic interaction with MDD but no significant interaction was founded [23], which strongly agreed with our current study. In contrast a study suggests that interaction between genes can also play an important role in onset of MDD [24]_,_ a study conducted in China on Han Chinese population the GSK-3Β gene SNP rs334535 was founded to be associated with MDD [25]. Thus, there is a need to investigate other GSK-3Β gene polymorphisms and the links between genetic polymorphisms and clinical features of psychiatric diseases.

Tryptophan hydroxylase (TPH) is responsible for serotonin and neurotransmitter synthesis and coded ahead by TPH1 and TPH2 both isoforms have same 70% sequence identity but the pattern of expression is different and both works on rate-limiting and the biosynthesis of serotonin. TPH1 isoform is predominant in periphery and the TPH2 in central nervous system [26]. Polymorphism of TPH1 gene is subjected for different kind of researches. Most known variation of this gene is A218C SNP (rs1800532) of intron 7 [27]. Different studies also showed that while expression of TPH1 gene the allele A of A218C play an important role [28]. In present study genotyping of TPH1 polymorphism in MDD patients and controls were not in agreement with the Hardy-Weinberg equilibrium (HWE). A study reported that genotype CC showed association to suicide attempts in individuals aged more than 65 years [29]. Our current study didn’t show any kind of association related to suicide and the association was not age related. Polymorphism of A218C TPH1 gene know to be linked with different disorder like Borderline personality disorder (BPD) in United States [27], suicidal behavior and anger-related personality behaviors [30], schizophrenia [31], bipolar disorder in Taiwan [32], Caucasian population [33] and depression in Finland [34]. Different association studies were conducted on TPH1 A218C polymorphism in Germany and China and were failed to find any association [35], there is lot of contradiction founded in this area.

TPH2 localized at (12q21.1) discovered after TPH1 play important role in neural tissue that’s why called brain specific isoform [36]. Compare to TPH1 it express more in brain that’s why it is suggested it has more significant role in brain region. Different studies reported variation of this genes leads to suicidal attempts, MDD and Seasonal Disorder [37,38], autism [39], and schizophrenia [40]. Finding of our current study TPH2 gene polymorphism (rs7305115) did not show any association with MDD. While comparing to previous studies the current finding is agreed with lots of studies. Association of allelic and genotypic frequencies in TPH2 in MDD patients [37,41], a study related to gender base do not show a significant result in TPH2 gene effect on susceptibility of MDD [42,43], another study target single locus and haplotype analysis didn’t show any association while targeting SNP rs7305115 in Japanese population [44].

Though the cellular level of DAT1 gene and this SNP rs40140 level have not demonstrated yet previous studies linked the polymorphism of unknown function to some psychiatric diseases [45]. So we conduct a study on genotypic distribution, allelic frequencies allele carrier frequencies on rs40184 same results was found no significant differences founded between groups case and control groups as well as in allelic frequencies, genotypic distribution and allele carrier frequencies in this particular SNP. Similar study carried out and founded no significant differences analyzing the same way but they observed more carriers C allele (CC+TT genotypes) in control when compared to patients so it suggest tendency toward association between genotype and TT homozygous this can be increased risk of MDD [46]. Previously another study reported that Russian population those reported with maternal rejection also had TT genotype of SNP rs40184 [47], founded to be settled with our study.

The SLC6A15 gene is based on a novel candidate gene responsible for MDD, but it remains unclear how the SLC6A15 genes alter the brain functional activities of the MDD patients. The SLC6A15 gene encoding a sodium-dependent branched amino acid transporter is the nearest annotated gene with a distance of about 287 Kb to the region of association [48]. In the present study we investigate the impact of polymorphism of SLC615 gene rs1545843 found no significant association between case and control samples. Recently, SLC6A15 was found to be associated with MDD, especially in a genome-wide association analysis, SNP rs1545843 documented with significant results [48]. Another study found the impact of rs1545843 in MDD patients on adrenocorticotropic hormone (ACTH) and cortisol levels was also shown and an associating of polymorphism with cognitive functions, like memories and continued attention, was already documented in these patients [49]. Although the proposed significance of these genes in the stress susceptibility documented in animal model research and potential links with MDD predisposition was recently investigated by GWAS [48]. The exact neurobiological role of this genetic polymorphism in the pathophysiologic phase of MDD and association of structural changes in specific brain regions in not clearly explained.

The genetic relationship between BDNF and MDD has been examined in a substantial amount of literature nevertheless the BDNF polymorphism is associated with an increased risk of depression reported in many studies [50], while some studies failed to find the association [51]. In our current study using single loci analysis BDNF rs6265 was not found to be correlated with occurrence of MDD studied in north-western population Pakistan. Several researchers reported the interaction between the BDNF gene and MDD stating that both pathophysiology and MDD diagnosis are extensively heterogeneous and thus this character makes them difficult to detect a susceptible gene for MDD [35,52]. There is several interpretation and discrepancies in addition MDD is complex disorder that is believed to be caused by effects of various genetic factors in the pathophysiology of the disease and gene-gen interactions are likely to contribute.

## Conclusion

We found no interaction between genetic risk loci and major depressive disorder but we still believe that the current study contains interesting results. It is further reported that the six candidate genes selected for this study supports the hypothesis that MDD has multifactorial behavior with a network of genetic interactions contributing small amount to the overall-risk of developing MDD. Most of the mental illnesses including MDDs have a number of complex phenotypes, with the patient cohorts too small and no findings have been consistently replicated. Moreover, the phenotypic effects of genetic variants identified so far are weak. It is more complex when we compare the impact of genetic variation on disease susceptibility and environmental factors. Despite these obstacles, the field of psychogenetic is still developing rapidly, and some new technological advances have been made (such as whole genome sequencing, GWAS). It is important to remember that genetic information will only provide additional information about one aspect of a psychiatric patient’s complex and personal history. In the end we recommend to study these six genes in more detail and explore other variants that may affect gene expression. GWAS studies and large scale analysis or additional work in different populations and appropriate analytical analysis are still needed to determine conclusively the etiology of MDD and other mental illnesses. Identifying these interactions will help to prioritize biological processes that require further study to better understand the etiology of MDD.

## Supporting information

S1 Table Questionnaire data. (XLSX)

S2 Table Demographics and clinical characteristics of MDD patients and control samples. (XLSX)

S3 Table HWE analysis. (XLSX)

S4 Table HWE calculations. (XLSX)
